# Draft Genomes of Two Strains of Flavobacterium Isolated from Lake Washington Sediment

**DOI:** 10.1128/genomeA.01597-14

**Published:** 2015-02-19

**Authors:** Tami L. McTaggart, Nicole Shapiro, Tanja Woyke, Ludmila Chistoserdova

**Affiliations:** a Department of Chemical Engineering, University of Washington, Seattle, Washington, USA; b DOE Joint Genome Institute, Walnut Creek, California, USA

## Abstract

We report sequencing the genomes of two new Flavobacterium strains isolated from Lake Washington sediment. From genomic contents, versatile lifestyles were predicted but not *bona fide* methylotrophy. With the availability of their genomic sequences, the new Flavobacterium strains present prospective models for studying microbial communities in lake sediments.

## GENOME ANNOUNCEMENT

When natural microbial communities from Lake Washington sediment are incubated under the atmosphere of methane, simple, semi-stable communities are formed, consisting of *bona fide* methanotroph species and of non-methanotrophic satellite species. Some of the most persistent satellites in such methane-fed microcosms are the Flavobacterium species, represented by multiple phylotypes (1). We isolated two Flavobacterium strains in pure cultures. Flavobacterium sp. 83 was isolated from an enrichment incubated with methylamine, as previously described (2). A single colony was selected, and axenic culture was obtained by restreaking onto fresh methylamine agar plates (2). Further growth tests revealed that methylamine was not required as a carbon source, and that the strain can grow on agar. The preferred medium is the nitrate mineral salts (NMS) medium (3). This culture forms transparent mucous colonies that look like droplets of water, likely due to the large amounts of the capsular polysaccharide produced. Cells are long and slender, with characteristic bulbous ends that look dark under the phase-contrast microscope. The strain is psychrophilic and does not grow at temperatures higher than 23°C. Flavobacterium sp. Fl was isolated from methane enrichment culture (low oxygen conditions; 1), by plating onto Nutrient Broth (NB) medium (Difco). Axenic culture was obtained by selecting a single colony, followed by multiple re-streaking onto fresh NB plates. This culture formed bright yellow, glossy, round colonies, the preferred medium being NB. The strain grows well at 30°C but not 37°C. Cells are short rods.

The draft genome sequences were generated at the DOE Joint Genome Institute (JGI), Walnut Creek, CA, USA using Pacific Biosciences (PacBio) sequencing technology (4). All general aspects of library construction and sequencing performed at the JGI can be found at http://www.jgi.doe.gov. The raw reads were assembled using HGAP (version 2.2.0.p1) (5). The final draft assemblies contain 1 and 21 contigs totaling, respectively, 3,790,620 and 5,284,058 bp in size.

Phylogenetically, both strains are novel showing less than 96% identity for the 16S rRNA gene with the closest named species, Flavobacterium psychrophilum. While more closely related to each other (97.1% 16S rRNA gene identity), they likely represent different species, based on the dramatic phenotypic differences and relatively low genome-genome similarity (only 10% of proteins show more than 90% identity, while 62% of proteins show more than 50% identity). As Flavobacterium sp. Fl possesses a much larger genome, 1,924 encoded proteins are unique to this strain (no homologs in Flavobacterium sp. 83 at 30% amino acid identity cutoff). 809 proteins are unique to Flavobacterium sp. 83. Both strains encode multiple functions for polysaccharide synthesis and degradation, and both encode gliding motility functions. Overall, versatile lifestyles can be predicted for both strains, but these do not include methylotrophy in a traditional sense (6). With the availability of their genomic sequences, the two new Flavobacterium strains present prospective models for studying microbial communities in lake sediments.

### Nucleotide sequences accession numbers.

The genome sequences have been deposited in GenBank under the accession numbers JQMS00000000 (Flavobacterium sp. 83) and JQJY00000000 (Flavobacterium sp. Fl).
